# Functionalized Electrospun Poly(Vinyl Alcohol) Nanofibrous Membranes with Poly(Methyl Vinyl Ether-Alt-Maleic Anhydride) for Protein Adsorption

**DOI:** 10.3390/ma11061002

**Published:** 2018-06-13

**Authors:** Mesbah Najafi, Joronia Chery, Margaret M. Frey

**Affiliations:** Department of Fiber Science & Apparel Design, Cornell University, Ithaca, NY 14853, USA; jc2566@cornell.edu (J.C.); mfw24@cornell.edu (M.M.F.)

**Keywords:** electrospinning, protein, adsorption, separation, polymer, nanofiber

## Abstract

In this work, electrospun poly(vinyl alcohol) (PVA) nanofiber membranes were functionalized by incorporating poly(methyl vinyl ether-alt-maleic anhydride) (poly(MVE/MA), PMA) for the selective adsorption of proteins. The capture performance was regulated by an optimizing buffer pH, PMA content, and protein concentration. Lysozyme was used as the model protein and a high adsorption capacity of 476.53 ± 19.48 was obtained at pH 6, owing to the electrostatic attraction between the negatively charged nanofibers and positively charged proteins. The large specific surface area, highly open porous structure, and abundant available carboxyl groups contributed to such high adsorption performance. Moreover, the nanofiber membranes exhibited good reusability and good selectivity for positively charged proteins. The obtained results can provide a promising method for the purification of proteins in small analytic devices.

## 1. Introduction

The purification and separation of proteins has become an essential prerequisite in present life science research, the pharmaceutical field, and the biomedical industry. Purified proteins are required for many experimental applications, such as structural studies, in vitro biochemical assays, biosensors and other protein-based analytical devices [1,2]. Several purification methods such as centrifugation, ultrafiltration, dialysis, sedimentation, adsorption, and chromatography have been developed. Among them, adsorption is of particular interest because of its cost-efficiency, time-efficiency, and ease in processing [3]. Traditionally, porous resin beads and gel microspheres have been used to capture selected species from protein mixtures [4]. The inherent problems of high pressure drops, high solution consumption, limited binding capacity, and low processing rates have restricted their use in large-scale applications [3,5]. 

Electrospun nanofiber membranes (ENMs) are excellent candidates for the adsorption of proteins because of their high specific surface area (SSA), highly tortuous porous structure, and robust mechanical properties [6]. In particular, these nanofibers facilitate chemical/biological functionalization, as the majority of the binding sites on their surface are external and accessible, which leads to a superb adsorption capacity. Several studies have been carried out to functionalize ENM surfaces for protein capture [7,8,9,10,11]. Ma et al. [7] functionalized electrospun poly(ether sulfone) (PSU) membranes with protein A/G, and the obtained nanofibers specifically adsorbed protein IgG with a binding capacity of 11.42 mg/g. Chiu et al. [9] hydrolyzed polyacrylonitrile nanofibrous membranes with sodium hydroxide and the obtained materials absorbed lysozyme with a capture capacity of 83.2 mg/g. Zhang et al. [10] covalently reacted a dye, Cibacron Blue F3GA, to the electrospun hybrid chitosan/nylon-6 nanofibrous membrane and the resultant adsorbents captured papain with a capacity of about 70 mg/g. Also, Schneiderman et al. [11] treated carbon nanofibers with nitric acid at 90 °C for 48 h to introduce carboxylate groups into a nanofiber surface for protein adsorption. The adsorption capacity of the nanofiber mats was about 10 times higher than that of the microfiber counterparts. Although these studies improved the adsorption performance of the nanofibers, the complexity of the chemical reactions, low adsorption efficiency, low binding protein capacity, and high cost of affinity ligands make them inappropriate for production in a large scale. 

In this study, we examined the effectiveness of functionalized electrospun PVA nanofiber membranes for protein adsorption. Our research group earlier demonstrated that PVA nanofibers can be functionalized by incorporating functional polymer poly(hexadimethrine bromide) (PB) with amine groups or poly(methyl vinyl ether-alt-maleic anhydride) (PMA) with carboxyl groups to create positively- and negatively-charged surfaces, respectively [12,13,14,15,16]. The surface charges of these nanofibers were confirmed previously by the thermally stimulated current (TSC) method [12]. Colangelo et al. utilized these nanofibers as on-chip biosensors for the immobilization of liposome and *E. coli* cells within microfluidic channels [13,14]. Also, Xiao et al. [12] recently used these charged nanofibers to selectively capture cationic dye methylene blue (MB) and acid red 1 (AR1) anionic dye from a dye mixture. 

Herein, we investigated the protein adsorption performance of PVA/PMA nanofiber membranes. This is the first time that such nanofibers were used for a protein study. Lysozyme (LYZ) was used as the model protein, as it has been widely used in industrial applications, such as food additives, antibacterial and anticancer agents [2]. The influence of various parameters, including pH, PMA content, and protein concentration, on the lysozyme capture were examined. Also, the selective adsorption of the membrane was examined in a LYZ/BSA binary solute system. The large SSA, highly tortuous open-porous structure, and abundant carboxyl groups of PVA/PMA nanofibers provided an excellent adsorption capacity, short equilibrium time, favorable reusability, and selectivity. The obtained results offer new insight into the development of functionalized nanofibers for protein adsorption and purification.

## 2. Materials and Methods

A highly hydrolyzed (98%) PVA with a molecular weight of 78,000 g/mol was purchased from Polysciences, Inc (Warrington, PA, USA). Poly(methyl vinyl ether-alt-maleic anhydride) (PMA, average *M*_w_ = 216,000 g/mol), Triton X-100 (p-tertiary-octylphenoxy polyethyl alcohol) were provided by Sigma-Aldrich (St. Louise, MO, USA). Lyophilized Lysozyme (LYZ) chicken egg white and bovine serum albumin (BSA) were purchased from Millipore Sigma (Burlington, NJ, USA). 

### 2.1. Electrospinning

A total of 10 wt % PVA was dissolved in 15 mL of DI-water in an oven at 95 °C for 4 h. PMA was dissolved in 10 mL of DI-water in an oven at 95 °C for 1.5 h and then mixed with PVA aqueous solution. The mass ratios of PVA/PMA were 90/10, 80/20, and 70/30 (*w*/*w*). Nonionic surfactant Triton X-100 was added to the spinning dope to reduce the surface tension of the water (X-100/DI-water = 0.5/99.5 *w*/*w*). The dope was loaded into a 5 mL BD plastic syringe equipped with a 21 gauge stainless needle. A high voltage of 18 kV was applied to the needle tip and a grounded aluminum collector was placed 20 cm away. The solution feed rate was maintained at 0.5 mL/h using a PHS ultrasyringe pump.

### 2.2. Heat Treatment

The as-spun nanofibers were thermally treated at 120 °C for 30 min under vacuum to increase the water stability of the membranes.

### 2.3. Adsorption Experiment

Protein solutions were prepared by dissolving a certain amount of lysozyme powder in a 0.01 M PBS buffer solution with various pH values. To investigate static adsorption, about 50 mg of PVA/PMA nanofiber membranes were immersed in a 30 mL lysozyme solution at different concentrations (0.2–1.0 mg/mL) and the mixture was shaken at 8 °C for a certain time. The protein concentration was measured on a PerkinElmer Lambda 35 UV−VIS spectrometer (Waltham, MA, USA) before and after the addition of nanofiber membranes. The adsorption capacities of the membranes were calculated using the following equation:

Q_e_ = V(C_0_ − C_e_)/m (1)

where Q_e_ is the adsorption amount (mg/g), V is the volume of the protein solution (mL), C_0_ and C_e_ are the initial and effluent concentrations of the protein solution (mg/mL), and m is the amount of adsorbent (g).

To test the dynamic adsorption, several layers of the PVA/PMA membranes were sandwiched into a plastic filter holder with a diameter of 1.3 mm. The filter holder was inserted into a 5 mL gastight Hamilton syringe (Reno, NV, USA) containing protein solution. The solution feed rate was maintained at 0.05 mL/min using a PHS ultrasyringe pump (Harvard Apparatus, Holliston, MA, USA). The effluent lysozyme solution was collected 1.5 mL at a time and the protein concentration of each effluent was obtained via the UV-VIS spectra.

To examine the membrane reusability, the adsorbed membrane was first eluted with 4.5 mL sodium hydroxide (1 M) using the syringe pump. Then, the nanofibers were rinsed by circulating 10 mL of DI water through the membrane to attain neutral conditions, and the adsorption experiment was repeated. To examine the protein removal, effluents were gathered during the base elution and the protein concentration of each effluent was obtained via the UV-VIS spectra.

### 2.4. Membrane Characterization

#### 2.4.1. FTIR Spectroscopy

All the membrane samples were analyzed by FTIR spectrometer (PerkinElmer-Frontier, Waltham, MA, USA) in the absorbance mode, at the range 4000–600 cm^−1^, with a scan resolution of 4 cm^−1^ and an average of 15 scans.

#### 2.4.2. FESEM Imaging

The morphology of the nanofibers was examined using a Zeiss 1550 Field Emission Scanning Electron Microscopy (Zeiss FESEM, Oberkochen, Germany). All samples were coated by a layer of Au/Pd and the images were obtained at an accelerating voltage of 3.0–5.0 kV.

##### Statistical Analysis

The diameter of the nanofibers was measured using the ImageJ software (1.52b National Institute of Health, Bethesda, MD, USA). An average of 50 individual fiber diameter measurements were reported for each sample.

#### 2.4.3. BET Surface Area

The Brunauer−Emmet−Teller (BET) surface area was characterized by measurement of the N_2_ adsorption–desorption isotherms at 77 °K with a surface area analyzer (Gemini VII 2390, Micromeritics Co., Norcross, GA, USA). 

### 2.5. Adsorption Selectivity

The capture selectivity of the membrane was examined using a dynamic adsorption test. To achieve this, 4 mg of LYZ and BSA were separately dissolved in 0.01 M PBS buffer solution with a pH value of 6. Then, 2 mL of these solutions was mixed together to create a 0.2 mg/mL BSA/LYZ solution (50/50 *w*/*w*). A dynamic adsorption test (details above) was conducted on the solution mixture and the protein effluent was gathered 1.5 mL at a time. LYZ solutions with various concentrations (0.1, 0.2, 0.3, 0.4 mg/mL) were also run with the protein mixture to examine the adsorption performance. The selectivity capacity was analyzed by sodium dodecyl sulfate polyacrylamide gel electrophoresis (SDS-PAGE) (12% gradient gel from Bio-Rad (Hercules, CA, USA) with standard Coomassie blue staining) before and after adsorption. 

## 3. Results and Discussion

### 3.1. Nanofiber Morphology and Structure

**Figure 1:**
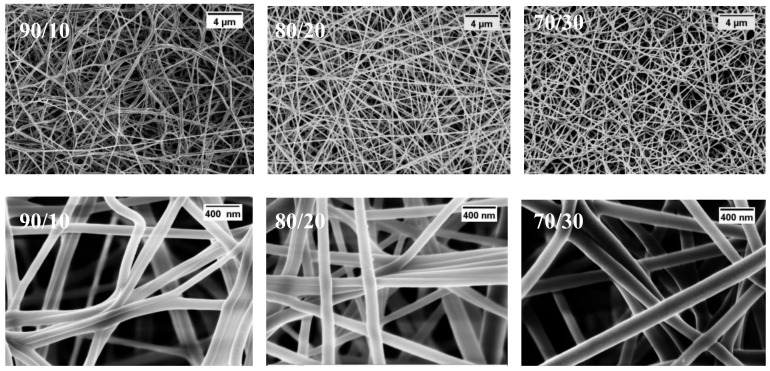
Fiber morphology of thermally-treated electrospun PVA/PMA membranes for different PMA contents. The morphology of pure PVA nanofibers can be found in our former study [15].

**Figure 2:**
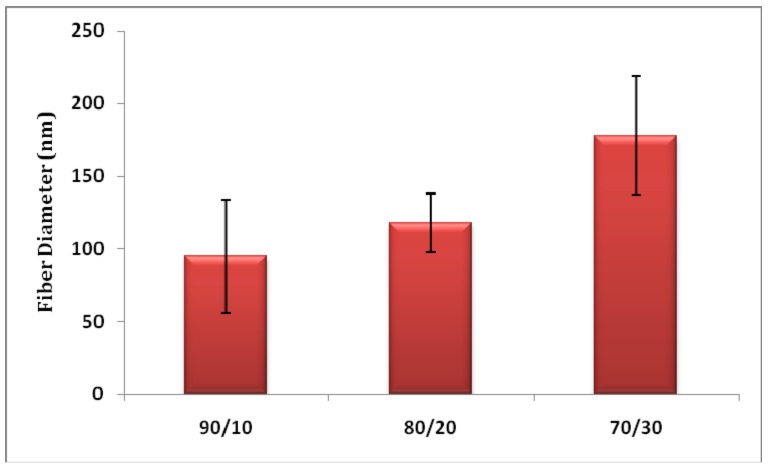
Nanofiber diameter of various PVA/PMA membranes after thermal treatment.

Figure 1 shows the morphology of heat-treated PVA/PMA nanofiber membranes at different PMA contents. As can be seen, the nanofibers are bead-free and have a uniform shape. The diameters of the nanofibers are compared in Figure 2. As can be seen, the fiber diameter increased from 89.14 ± 41.12 to 177 ± 40.51 nm, when the PMA content increased from 10 to 30%. It is known that a higher *M*_w_ polymer increases the viscosity of the electrospinning solution and results in a larger fiber diameter [17]. As the PMA has a considerably higher *M*_w_ than the PVA, the higher PMA contents can increase the solution viscosity and fiber diameter. 

The BET surface area of PVA/PMA (80/20) nanofibers was 13.056 ± 0.197 m²/g, which is significantly high for materials adsorption [4]. As a high protein adsorption capacity was obtained for the 80/20 blend (details in *effect of PMA content*), this measurement was performed on that sample. The pore size distribution of the fiber webs was measured with a capillary flow porometer. The mean flow pore diameter of the nanofiber membranes was 221.2 ± 136.5 nm and the maximum pore size was 384.5 nm. Such a high surface area and highly open porous structure provide the possibility for the nanofibers to adsorb proteins.

### 3.2. Adsorption Performance

**Figure 3:**
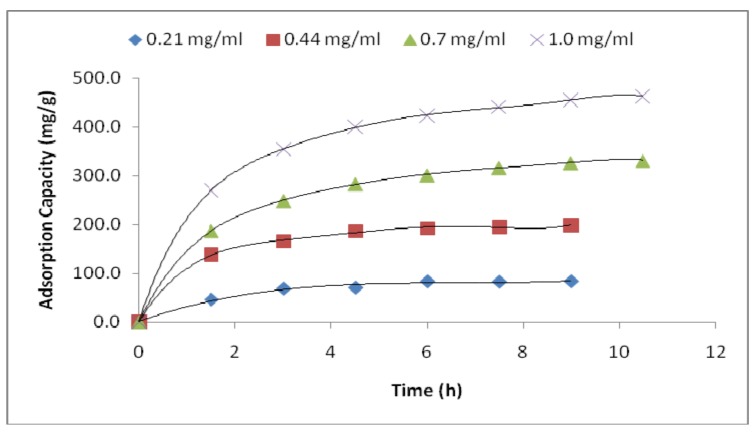
Static adsorption performance of the PVA/PMA (80/20) nanofiber membrane at different lysozyme concentrations (pH = 6) as a function of time.

Figure 3 and Table 1 display the adsorption performance of PVA/PMA membranes at different lysozyme concentrations. As we obtained a higher adsorption capacity for the PVA/PMA (80/20) ratio (details in *effect of PMA content*), we used this nanofiber to examine the adsorption performance. The adsorption amount first increased rapidly with the time and then reached equilibrium within about 6 to 10 h, depending on the protein concentration. This is because initially there were many available adsorption sites on the nanofiber surface for lysozyme capture, resulting in the rapid migration of proteins from the solution on the fiber surface. Once the membrane and the protein have been in contact long enough, equilibrium will be established between the amount of proteins adsorbed on the fibers and the amount of proteins in the solution and the adsorption capacity reaches a plateau. The high adsorption capacity was 476.53 ± 19.48 mg/g, which was related to the 1.0 mg/mL lysozyme concentration. It is interesting that such a high adsorption capacity was obtained simply through the incorporation of a functionalized polymer into PVA nanofibers. Wang et al. [4] produced carboxyl-surface-functionalized nanofibers by combining electrospinning and the in-situ graft polymerization of PVA and maleic anhydride (MAH). They reported a lysozyme adsorption capacity of about 200 mg/g for the nanofibers with a 320 nm diameter and 3.2 g/m^2^ BET surface area. In comparison, in our study, we initially incorporated a high *M*_w_ (216 KD) PMA polymer into the electrospun PVA nanofibers and our capture capacity was nearly two times that of the previously reported data. This higher capacity can be ascribed to the abundant adsorbent carboxyl groups (more details in the FTIR analysis), 270% smaller fiber diameter, and 400% higher specific surface area of the membranes.

### 3.3. Optimization of Adsorption Conditions

#### 3.3.1. Effect of pH

**Figure 4:**
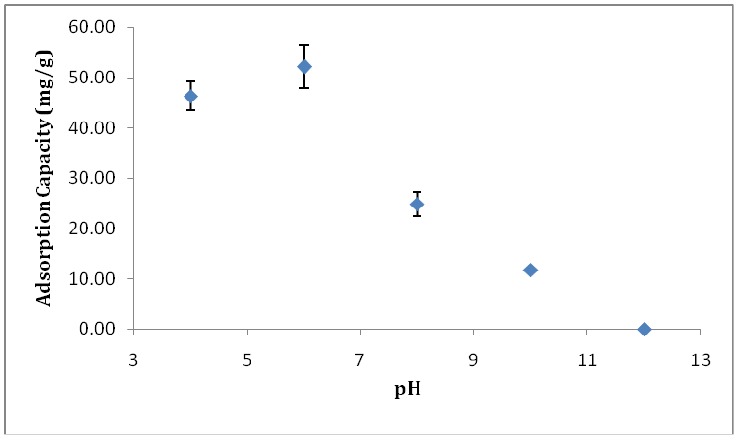
Effect of pH on the static adsorption capacity of PVA/PMA (80/20) membranes for lysozyme solutions (0.2 mg/mL).

**Figure 5:**
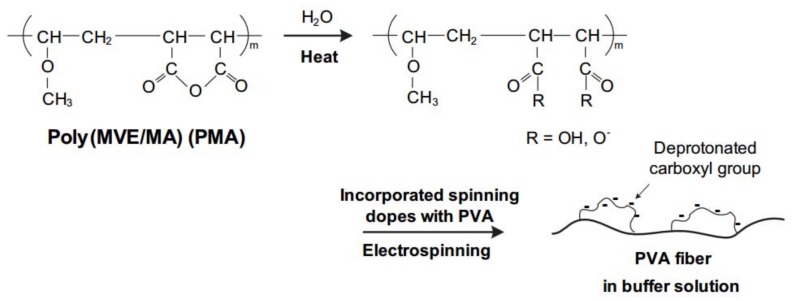
Scheme of negative surface charges of PVA/PMA nanofibers. The pH = 4 is necessary to produce the hydrolysis. (Reprinted from ref. [15] with the permission of Elsevier).

The buffer pH plays a considerable role in protein adsorption, as it can regulate the surface charge of both nanofibers and proteins. Figure 4 displays the adsorption capacity of PVA/PMA nanofiber membranes at different pH values. A low protein concentration (0.2 mg/mL) was chosen for this experiment to analyze the results faster. As can be seen, at pH 12 the membrane did not adsorb lysozyme. However, when the pH is less than the lysozyme isoelectric point (LYZ IP), the adsorption capacity increased and reached to the maximum at pH 6. This phenomenon can be explained by the surface charge of nanofibers and proteins. As the LYZ IP is about 10.8, the positive charges of the protein will increase when decreasing the pH from 10 to 6. Moreover, PMA has functional groups maleic anhydride, which is hydrolyzed into carboxyl groups once in contact with water (Figure 5). These carboxyl groups ionized into COO- at pH > 4 (pKa (-COOH) ~4 [18]) which results in negative surface charges on the nanofibers. The electrostatic attraction between the positively-charged proteins and the negatively-charged nanofibers resulted in the higher adsorption capacity at a lower pH. 

**Figure 6:**
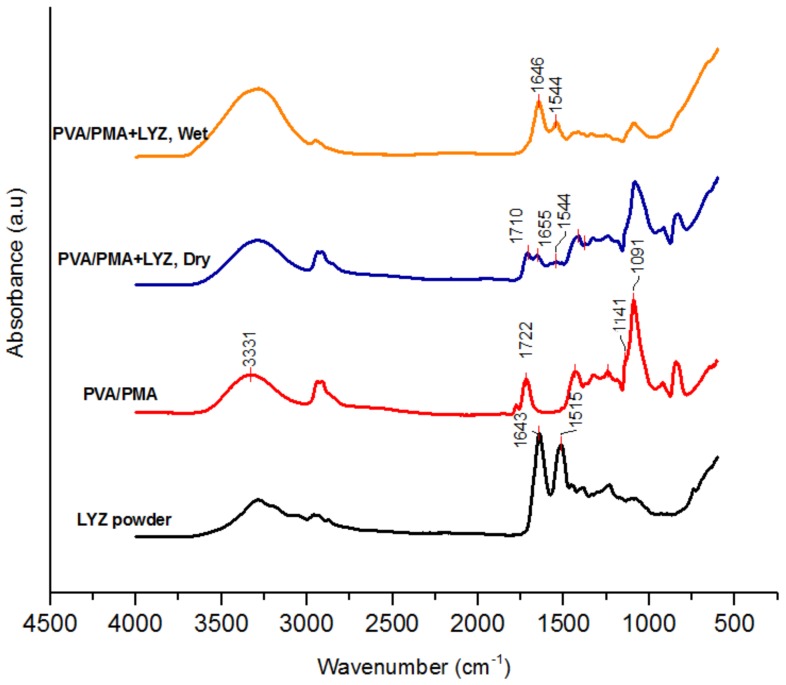
FTIR spectra of PVA/PMA (80/20) membrane before and after lysozyme adsorption.

Figure 6 shows the FTIR spectra of the PVA/PMA (80/20) membranes before and after lysozyme adsorption. The spectrum of the lysozyme powder is also displayed for comparison. The amide peaks I and II at about 1643 and 1515 cm^−1^ related to LYZ [19] can be clearly seen at PVA/PMA + LYZ. This confirms that the protein was adsorbed on the surface of the nanofiber membranes. Moreover, the adsorption peak at 1722 cm^−1^ related to the carbonyl group of PMA was not present on the spectrum of the adsorbed (wet) membranes. Also, the peaks at 1091 and 3331 cm^−1^ related to the C-O and O-H groups of PVA had a lower and a higher intensity compared to those of the pristine membrane. This might be related to the water inside the membrane, which can affect the FTIR peak resolution. The water can form hydrogen bonding with the hydroxyl and amide functional groups of the polymers influencing the shape/adsorption of the peaks. When the membrane was dried completely, those adsorption peaks reappeared on the spectrum. The obtained pH/FTIR results for 0.2 mg/mL can be applied to higher lysozyme concentrations. 

#### 3.3.2. Effect of PMA Content

The PMA content can considerably influence the protein adsorption, as it affects the number of carboxyl groups on the nanofiber surface. Table 2 shows the static lysozyme adsorption of PVA/PMA membranes at different PMA contents. Increasing the PMA from 10 to 20% raised the adsorption capacity from 44.75 ± 2.53 to 80.07 ± 3.71 mg/g by ca. 79%. This addition also decreased the equilibrium time from 6 to about 4 h. Such adsorption increase can be explained by the fact that at a higher amount of PMA, more adsorbent carboxylic groups are available on the membrane surface. Once in contact with the buffer solution, these carboxylic groups are ionized into carboxylate groups, resulting in a faster and higher capture of positively-charged proteins. The addition of 30% PMA did not significantly increase the adsorption capacity due to the larger fiber diameter (Figure 2) and thus the smaller specific surface area of that membrane. As a high adsorption capacity was observed for the 80/20 fiber, this sample was used for subsequent characterization tests. 

**Figure 7:**
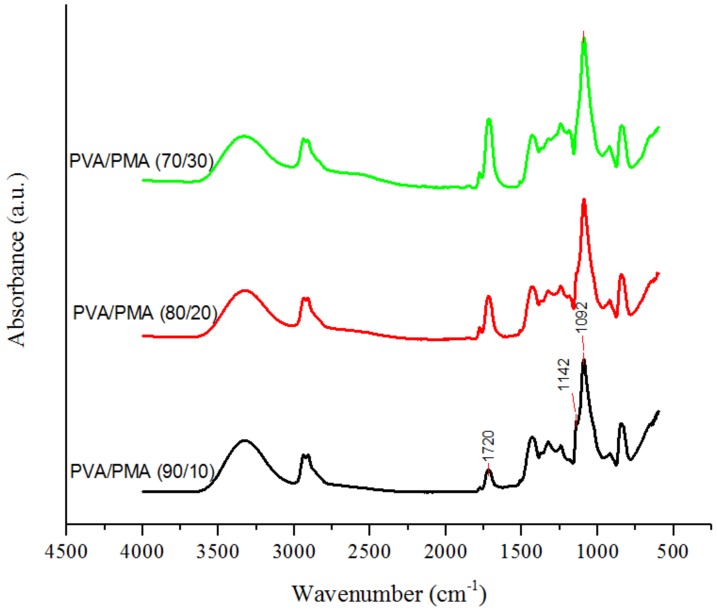
FTIR spectra of thermally treated PVA/PMA nanofiber membranes for different PMA contents.

FTIR was conducted on the membranes to confirm the increasing amount of PMA in the nanofibers. Figure 7 displays the FTIR spectra of the samples for different amounts of PMA. The absorbance peak at 1720 cm^−1^ is attributed to the carboxylic group of PMA. Also, the absorption peak at 1142 cm^−1^ related to C-O-C stretching is for crosslinking between PMA and PVA during heat treatment. The crosslinks are formed through an esterification reaction between maleic anhydride groups of PMA and the hydroxyl groups of PVA [12,15]. Table 3 shows the intensity ratios of the functional groups. The C-O-C(stretching)/C-O(stretching) ratio decreased and C=O(stretching)/C-O(stretching) ratio increased with the PMA content, indicating more available carboxylic groups (-COOH) on the 80/20 or 70/30 nanofiber surface for lysozyme capture. 

### 3.4. Membrane Reusability

**Figure 8:**
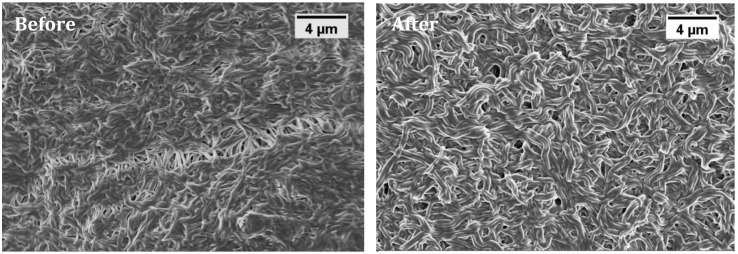
SEM images of PVA/PMA (80/20) nanofiber membrane adsorbed lysozyme before and after NaOH elution.

Membrane reusability is the use of existing membranes for multiple cycles before replacing them. Reusing membranes can increase the operational flexibility and reduce material and labor costs. To investigate this, the adsorbed membrane was first eluted with sodium hyroxide (1 M), and was then rinsed with DI water using a syringe pump. Table 4 reveals the lysozyme concentration of the PVA/PMA (80/20) membrane before and after sodium hydroxide elution. As it can be seen, the base removed most of the proteins from the membranes. Interestingly, the adsorption capacity of the membrane before and after the elution was almost the same, indicating the effectiveness of the recycling process. This can be explained by the fact that the base increased the pH around the membrane, weakening the electrostatic interaction between the charged nanofibers and the charged protein. When pH > IP, both the lysozyme and nanofibers are negatively charged and the repulsive force between them results in the complete removal of the protein from the membrane surface. Figure 8 displays the morphology of the nanofiber membranes before and after elution. As can be seen, the nanofibers were a little bit swollen after the elution, due to the water absorbed by the PVA. Nevertheless, the physical structure of the fibers remained stable after elution due to chemical crosslinks between the polymers during heat treatment. The obtained results would enhance the practical applications of PVA/PMA membranes for protein separation.

### 3.5. Adsorption Selectivity

**Figure 9:**
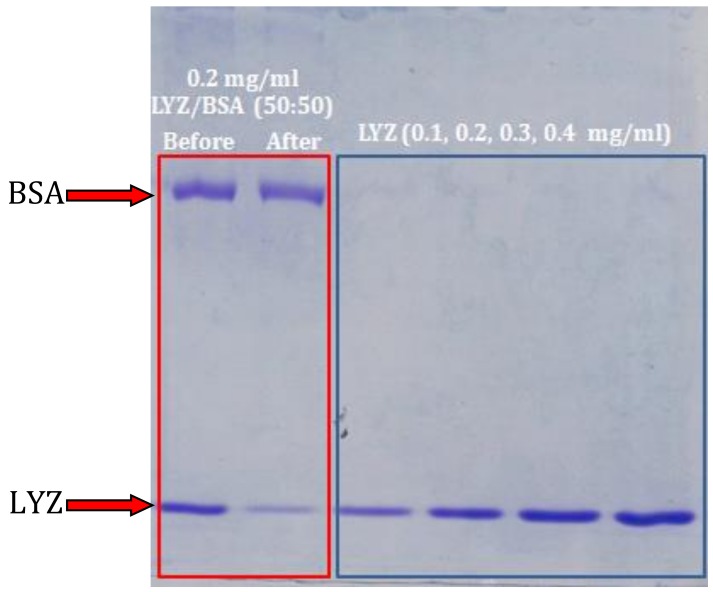
SDS-PAGE analysis for the purification of LYZ solutions and LYZ/BSA mixture (Buffer pH = 6).

Adsorption selectivity is one of the important features of polymer membranes. It indicates the ability of a membrane to capture a specific product from a mixture. The selectivity of PVA/PMA membranes for protein adsorption was examined by SDS-PAGE. LYZ (IP of 10.8) and BSA (IP of 4.8) were taken as positively- and negatively-charged model proteins, respectively. To examine the adsorption performance, LYZ solutions with various concentrations were also run with the protein mixture. As illustrated in Figure 9, it is clear that the band color of the lysozyme became lighter after they were adsorbed by the membranes. The band intensity was even lower than that of the 0.1 mg/mL LYZ solution, indicating that the PVA/PMA membrane mainly adsorbed the LYZ. At pH 6, the charges of the BSA and PVA/PMA nanofibers were both negative, while the charge of LYZ was positive. Thus, the nanofibers repelled the BSA and attracted the LYZ, leading to selective protein adsorption from a mixture. This result can be extended to other positive and negative proteins, increasing the versatility of PVA/PMA ENM for protein purification applications.

## 4. Conclusions

In this study, PMA polymer was incorporated into electrospun PVA nanofibers to create composite membranes for protein adsorption. The effects of various parameters such as buffer pH, PMA content, and protein concentration on the adsorption performance of the membranes were investigated. A high adsorption capacity of about 476 mg/g was obtained for the PVA/PMA (80/20, *w*/*w*) membrane at 6 pH buffer. This high capacity was due to the combined effects of the highly-specific surface area, highly porous structure, and abundant carboxyl groups of the nanofibers. By tailoring the pH, the negatively-charged nanofibers selectively adsorbed positively-charged proteins from a protein mixture. Also, the results showed that the membranes can be regenerated/reused with no significant effect on the capture capacity. The PVA/PMA nanofibers exhibited great potential for the production of effective and low cost membranes for protein purification. Future work will focus on the utilization of these nanofibers for the separation of proteins in microfluidics analytical systems. 

## Figures and Tables

**Table 1:** Static adsorption capacities (Q_e_) of PVA/PMA (80/20) membranes at different lysozyme concentrations.

LYZ Conc. (mg/mL)	0.21	0.44	0.7	1.0
**Qe (mg/g)**	86.29 ± 3.89	207.88 ± 13.12	326 ± 7.35	476.53 ± 19.48

**Table 2:** Static adsorption performance of PVA/PMA nanofiber membranes for different PMA contents (LYZ Conc.: 0.2 mg/mL).

PVA/PMA	C	Q_e_
*w*/*w* (%)	(mg/mL)	(mg/g)
70:30	0.22	81.94 ± 2.09
80:20	0.21	80.07 ± 3.71
90:10	0.21	44.75 ± 2.53

**Table 3:** Adsorption ratios of C=O/C-O and C-O-C/C-O in PVA/PMA nanofibers at different PMA contents.

PVA/PMA	C=O/C-O	C-O-C/C-O
90/10	0.16	0.54
80/20	0.29	0.46
70/30	0.43	0.38

**Table 4:** Lysozyme concentrations before and after the NaOH elution of PVA/PMA (80/20) membranes in a dynamic adsorption test.

Solution	LYZ	Adsorption
Type	Conc. (mg/mL)	Capacity (mg/g)
Pristine LYZ solution	0.18	0.0
Effluent—1st LYZ Adsorption	0.03	55.1
Effluent—Base Elution	0.17	14.1
Effluent—2nd LYZ Adsorption	0.02	59.6
